# SAA restricts T cell mediated anti-tumor immunity by limiting antigen presentation in lung cancer

**DOI:** 10.3389/fimmu.2025.1735479

**Published:** 2025-12-19

**Authors:** Mei Huang, Run Shi, Cong Xu, Yihan Zhang, Xiaoyue Du, Shaodi Wen, Chunbin Wang, Feng Jiang, Guoren Zhou, Xin Wang, Bo Shen

**Affiliations:** 1 Department of Oncology, Jiangsu Cancer Hospital & Jiangsu Institute of Cancer Research & The Affiliated Cancer Hospital of Nanjing Medical University, Nanjing, China; 2 Department of Oncology, Yancheng Third People’s Hospital, Yancheng, China; 3 The Sixth Affiliated Hospital of Nantong University, Nantong, China; 4 Department of Oncology, First Affiliated Hospital of Nanjing Medical University, Nanjing, China; 5 Department of Thoracic Surgery, Jiangsu Cancer Hospital & Jiangsu Institute of Cancer Research & The Affiliated Cancer Hospital of Nanjing Medical University, Nanjing, China; 6 Department of Oncology, Affiliated Huishan Hospital of Xinglin College, Nantong University, Wuxi Huishan District People’s Hospital, Wuxi, China; 7 Department of Oncology, Huai’an Hongze District People’s Hospital, Huaian, China

**Keywords:** APC, cancer immunotherapy, CD36, lung cancer, SAA

## Abstract

**Background:**

Serum amyloid A (SAA), an acute-phase pro-inflammatory protein, is overexpressed in several cancers and is involved in shaping pro-tumor responses. We have previously reported that lung cancer stem cells secrete SAA, which contributes to tumor progression by inhibition of T_H_1 immunity. Here, we extended our studies to examine the mechanism of SAA mediated immunosuppression in both antigen-presenting cells (APCs) and the subsequent activation of T cells.

**Methods & results:**

Using *ex vivo* co-culture systems and *in vivo* mice models, we found that SAA impaired dendritic cell and macrophage activation and drove macrophages toward an M2 phenotype with reduced antigen presentation. Lung cancer cells overexpressing SAA also consistently showed impaired CD8^+^ T cell infiltration and cytotoxicity, while SAA neutralization were efficient at enhancing CD8^+^ T cell activation and response to anti-tumor immunity. Mechanistically, we found that the immunosuppressive phenotype induced by SAA on APCs is mediated in part by CD36. Critically, inhibiting SAA by neutralization antibody recovered APC activity and enhanced T cell-dependent tumor control.

**Conclusion:**

our results identify SAA as an important immunosuppressive mediator in the tumor microenvironment, implying that the SAA neutralizing antibody may be a potential target for the improvement of lung cancer immunotherapy.

## Introduction

Lung cancer is a major cause of cancer death in the world, and many patients are diagnosed at an advanced stage, of whom the overall survival (OS) is poor (1, 2). Recent years, immunotherapy and particularly immune checkpoint inhibitors (ICIs), have provided great improvement for some patients, nevertheless, a considerable number of lung cancer patients remain unresponsive to ICIs, and the mechanisms of the resistance are still not well defined (3, 4).

The antitumor immune response is fundamentally dependent on the effective activation and functioning of APCs, including DCs and macrophages. These cells play a crucial role in capturing, processing, and presenting tumor antigens to T lymphocytes (5). The generation and sustained activity of cytotoxic T cell responses, which are vital for controlling and eliminating tumors, also hinge on the efficient antigen presentation by APCs. However, dysfunction or suppression of APCs within the tumor microenvironment can lead to impaired T cell activation and immune evasion, ultimately contributing to tumor progression and resistance to immunotherapies (6).

Tumor cells including aggressive subpopulations like cancer stem cells (CSCs) can secrete immunosuppressive cytokines and soluble factors that blunt APC function and downstream T cell activation (7–9). For example, tumor-derived IL-10 and TGF-β drive DCs into a tolerogenic state, reducing MHC-II and co-stimulatory molecule expression while upregulating inhibitory ligands (e.g. PD-L1), which together impair effector T cell priming (10). Tumor-produced PGE_2_ (via COX-1/2) and IL-6 can likewise inhibit DCs differentiation through STAT3 signaling, skewing DCs to produce more IL-10 but less IL-12, thereby promoting regulatory or T_H_2 responses over T_H_1 cytotoxic immunity (11, 12). In addition, tumors recruit and polarize macrophages into immunosuppressive tumor-associated macrophages (M2 TAMs) by secreting factors such as CCL2, IL-8, or M-CSF (13). These TAMs release IL-10 and TGF-β, further suppressing CD8^+^ T-cell activation and fostering a tumor-permissive immune microenvironment (13).

Serum amyloid A (SAA) is an acute-phase protein that is highly expressed in various inflammatory conditions and cancers (14, 15). Our previous research demonstrated that SAA, secreted by cancer stem cells, could suppress anti-tumor immunity in lung adenocarcinoma by inhibiting T_H_1 polarization and promoting tumor fibrosis, thereby limiting the efficacy of immune checkpoint blockade (16). However, the detailed cellular mechanisms by which SAA modulates the anti-tumor immune response remain to be elucidated.

In current study, we investigated the effect of SAA on APCs and its role in priming T cells in anti-tumor immunity. By introducing both *ex vivo* co-culture and *in vivo* tumor models, we explored the role of SAA on the maturation and polarization of DCs and macrophages, and their antigen presentation capabilities and subsequent T cell activation. This impairment of antigen presentation disrupts T cell activation, leading to reduced T cell–mediated cytotoxicity. Our study provides novel insights into the immunosuppressive function of SAA and indicating SAA neutralization might become a new approach for immunotherapy.

## Materials and methods

### Bioinformatic analysis

Data acquisition and processing: Two single-cell RNA (scRNA) sequencing datasets were downloaded from the TISCH2 database, comprising samples from five patients with primary non-small cell lung cancer (NSCLC) tissue (EMTAB6149) and three patients with brain metastatic cancers (GSE143423) (17, 18). Data re-analysis was performed using the “Seurat” (v4.4.0) package. The filtered gene expression matrix was normalized using the “NormalizeData” function. Principal component analysis (PCA) identified 5,000 highly variable genes (HVGs), and the number of principal components was set to 30. Batch effects were removed using the “RunHarmony” method from the “harmony” package. The original cell clustering was retained. Malignant cells were further divided into SAA1-positive (SAA1+Malig) and SAA1-negative (SAA1-Malig) malignant epithelial cells based on unique molecular identifier (UMI) values (whether UMI > 0 or not). Myeloid cells were further divided at a resolution of 1 and annotated into monocytes (FCN1, AIF1, LST1, LYZ), M1 macrophages (CD80, CD86, CXCL10), M2 macrophages (CD163, CD68, MRC1, CCL18), conventional DC type 1 (CLEC9A, XCR1, BTLA, CD226), conventional DC type 2 (CD1C, FCER1A), and plasmacytoid DCs (IRF4, LILRA4, CLEC4C). Cluster biomarkers were identified using the “wilcoxauc” function (“presto” package) and referenced from the CellMarker 2.0 database (19–21).

Immune functional profiling and cellular trajectory analysis: The assessment of immune function was conducted using 13 previously published immune-related gene sets and the “UCell” algorithm for computation (22, 23). The 13 immune-related functional scores assessed include: APCs co-inhibition, APC co-stimulation, chemokine receptor (CCR), immune checkpoint, cytolytic activity, human leukocyte antigen (HLA), inflammation promotion, major histocompatibility complex (MHC) class I, parainflammation, T cell co-inhibition, T cell co-stimulation, type I interferon response, and type II interferon response. Pseudotime trajectory analysis was conducted using “monocle” package (v2.26.0) to reconstruct cellular differentiation trajectories based on single-cell transcriptomic data of malignant epithelial cells (24). Intercellular communication was inferred with “CellChat” package (v2.2.0), which systematically analyzes ligand–receptor interactions between cell populations among tumor microenvironment (25). All analyses were performed using default parameters.

Differential expression and pathway enrichment: Differential gene expression analysis between SAA1-positive and SAA1-negative malignant cells was performed using the “Libra” package (v1.0.0), applying the “run_de” function with “de_family” set to “singlecell” and “de_method” set to “wilcox” (26). The selection criteria for differential expressed genes were set as average log fold change (avg_logFC) greater than 0.25 and adjusted p-value (p_val_adj) less than 0.05. To explore the key biological functions associated with SAA1^+^ malignant cells, 50 hallmark gene sets were collected from the Molecular Signatures Database (MSigDB) (27). To minimize bias from a single gene set enrichment analysis method, enrichment results from six approaches (AUCell, UCell, singscore, ssgsea, JASMINE, and viper) were integrated using a robust rank aggregation (RRA) algorithm (RRA score < 0.05) implemented via the “irGSEA” and “RobustRankAggreg” packages (28, 29).

### Cell line and transfection, primary sample preparation, and *ex vivo* experiments

Human A549, THP1 and murine LLC cell lines were obtained from the National Collections of Authenticated Cell Cultures (Shanghai) and maintained in recommended media. All cell lines were confirmed to be mycoplasma-free. To generate cells with altered SAA expression, LLC cells were transduced with adeno-associated virus vectors to stably overexpress full length human SAA1 or a control sequence respectively. Stable transductants were selected with 2 µg/mL puromycin and verified for SAA expression by RT-qPCR. For preparation of tumor antigens, tumor cell lysates were prepared by three freeze–thaw cycles for use as soluble tumor antigens.

Fresh tumor specimens were obtained from patients with lung adenocarcinoma and immediately placed on ice following surgical resection. Tissues were transferred into sterile tubes containing primary tissue storage solution (K601005, BioGenous) and transported to the laboratory on ice within 24 hours to maintain sample integrity. Upon arrival, samples were washed in tissue storage buffer with antibiotic for 30 minutes at room temperature on an orbital shaker. Tumor tissues were then minced into small fragments (approximately sesame seed-sized) and subjected to enzymatic digestion (130-095-929, Miltenyi Biotec) at 37°C for 40–50 minutes in a thermoshaker. The resulting cell suspension was pipetted thoroughly to ensure complete dissociation and filtered through a 70 μm cell strainer. Isolated cells were resuspended in Matrixgel (40183ES10, Yeasen Biotechnology) and plated into 6-well plates. During the initial 48 hours, primary cells were cultured in lung cancer organoid medium (K2138-LA, BioGenous) supplemented with 10 μM ROCK inhibitor (HY-10071, MedChemExpress) for the first 24 hours only. The culture medium (without ROCK inhibitor) was subsequently refreshed every 48 hours (16).

Peripheral blood mononuclear cells (PBMCs) were isolated from heparinized human blood samples (healthy donors or patients) by density gradient centrifugation. Whole blood was diluted 1:1 with phosphate-buffered saline (PBS) and layered over Ficoll-Paque PLUS (Cytiva) in centrifuge tubes. Samples were centrifuged at 400 × g for 30 min at room temperature with no brake. The mononuclear cell layer at the plasma–Ficoll interface was collected, transferred to a fresh tube, and washed twice with PBS (300 × g, 10 min) to obtain PBMCs (30). PBMCs were resuspended in RPMI-1640 medium with 10% FBS and 2 mM L-glutamine at 2×10^6^ cells/mL, then plated in cell culture dishes. Cells were incubated for ~12 h at 37 °C to allow monocytes to adhere to the plastic. Non-adherent cells (enriched in lymphocytes) were gently removed by washing with warm PBS and saved for T cell preparations. Adherent monocytes and non-adherent PBMCs were cultured in complete RPMI medium containing cytokines for macrophage, dendritic cell differentiation and/or treatment, including recombinant human M-CSF (11792-HNAH, SinoBiological), GM-CSF (10015-HNAH, SinoBiological), IL4 (11846-HNAE, SinoBiological), IL6 (10395-HNAH, SinoBiological), TNF (10602-HNAE, SinoBiological), IL2 (11848-HNAH1-E, SinoBiological), CD3 antibody (16-0037-81, ThermoFisher), CD28 antibody (14-0289-82, ThermoFisher), SAA antibody (924903, R&D), rec. SAA1 (300-53, PeproTech), SMS121/CD36 inhibitor (HY-163541, MCE) and TLR2/4 inhibitor (NBP2-26245, Novus). Fresh medium with cytokines was replenished every 2 days. For the following co-culture experiments details in the previously published article from our research group (16). Each condition was assayed in at least triplicate wells.

For co-culture experiments, after removal of Matrigel and preparation of single-cell suspensions, PBMCs were co-cultured with tumor organoids in the presence of recombinant SAA, control, or SAA-neutralizing antibody (α-SAA, anti-SAA1 & 2). Briefly, tumor specimens were cultured to establish organoids. PBMCs were isolated from peripheral blood and, according to experimental groups, either pretreated or activated, with cell differentiation induced by rhGM−CSF, rhIL−6, rhIL−1β and/or TNFα (depending on cell type) and T−cell activation performed using α−CD3/α−CD28−coated plates supplemented with rhIL−2. On Day 0 organoids were prepared and PBMCs were thawed from liquid nitrogen and recovered in culture; on Day 3 organoid−derived single cells were co−cultured with treated PBMCs a with continued cytokine supplementation (until Day 5, for cytokine measurement); on Day 6 cells and culture supernatants were collected for flow cytometry immune phenotyping and other downstream molecular and functional analyses.

### Mouse tumor model

All animal experiments were approved by the Nanjing Medical University Animal Care and Use Committee and conducted in specific pathogen-free conditions. Female C57BL/6 mice (6–8 weeks old) were used for tumor implantation. After 18 days, mice were anesthetized with isoflurane and injected subcutaneously in the right flank with 10^6^ tumor cells in 100 µL of serum-free RPMI (1:1 mix with Matrigel, 40183ES10, Yeasen Biotechnology) to establish tumors. Tumor growth was measured every 3 days using calipers, and tumor volume was calculated as (length×width^2^)/2. Mice received intraperitoneal injections of anti-mouse PD-1 antibody (BE0146, BioXCell, 200 µg/mouse, 3 doses) and SAA neutralization antibody (abinScience, HY110010, 200 µg/mouse, 3 doses)/isotype control depends on treatment group. Tumor size was monitored over time, and mice were sacrificed when any tumor reached ~1.5 cm in diameter or showed ulceration, in accordance with humane endpoints. Tumors were processed with tumor dissociation kit (mouse) (130-096-730, Miltenyi Biotec) following official instructions for downstream analyses.

### Flow cytometry and immunohistochemistry analysis

For flow cytometry, single-cell suspensions from tumor tissue or *ex vivo* cultures were prepared by enzymatic digestion. Cells were stained using viability dyes, CellTracer, Fc-block, and specific antibodies, details in an antibody list (**Supplementary Table 1**). For supernatant cytokine measurements, a CBA kit was introduced (Cytokines Multiplex Detection Kit, Twelve‑Plex, NMPA, 100 tests/kit; P110100403, from JIANG XI Cellgene Biotechnology Co., LTD.) following official instruction. Briefly, Dilute standards to gradients. Mix capture microspheres, then combine with culture supernatants/standards and detection antibody. Incubate in dark. Wash, resuspend, and run on flow cytometer. Flow cytometry was performed on BD FACSCelesta; data were analyzed with FlowJo and FCS Express software, details in our previous work (16, 31).

For IHC, Paraffin-embedded tumor sections (4 µm) were deparaffinized, rehydrated, and subjected to citrate buffer–based antigen retrieval. After blocking endogenous peroxidase and nonspecific binding, slides were incubated overnight at 4 °C with primary antibodies (Ki67, ab15580, Abcam; SAA, 16721-1-AP, Proteintech; Cd8, 85977-4-RR, Proteintech), followed by HRP-conjugated secondary antibodies. Staining was visualized using DAB substrate and counterstained with hematoxylin before mounting.

### Data analysis and statistics

Data are expressed as mean ± SD. Statistical analyses were conducted using GraphPad Prism 9.5.1. Comparisons were made using one-way ANOVA with Tukey’s *post-hoc* test. Differences with *P* < 0.05 were considered significant.

## Results

### SAA expression correlates with an immunosuppressive immune landscape in LUAD

**Figure 1:**
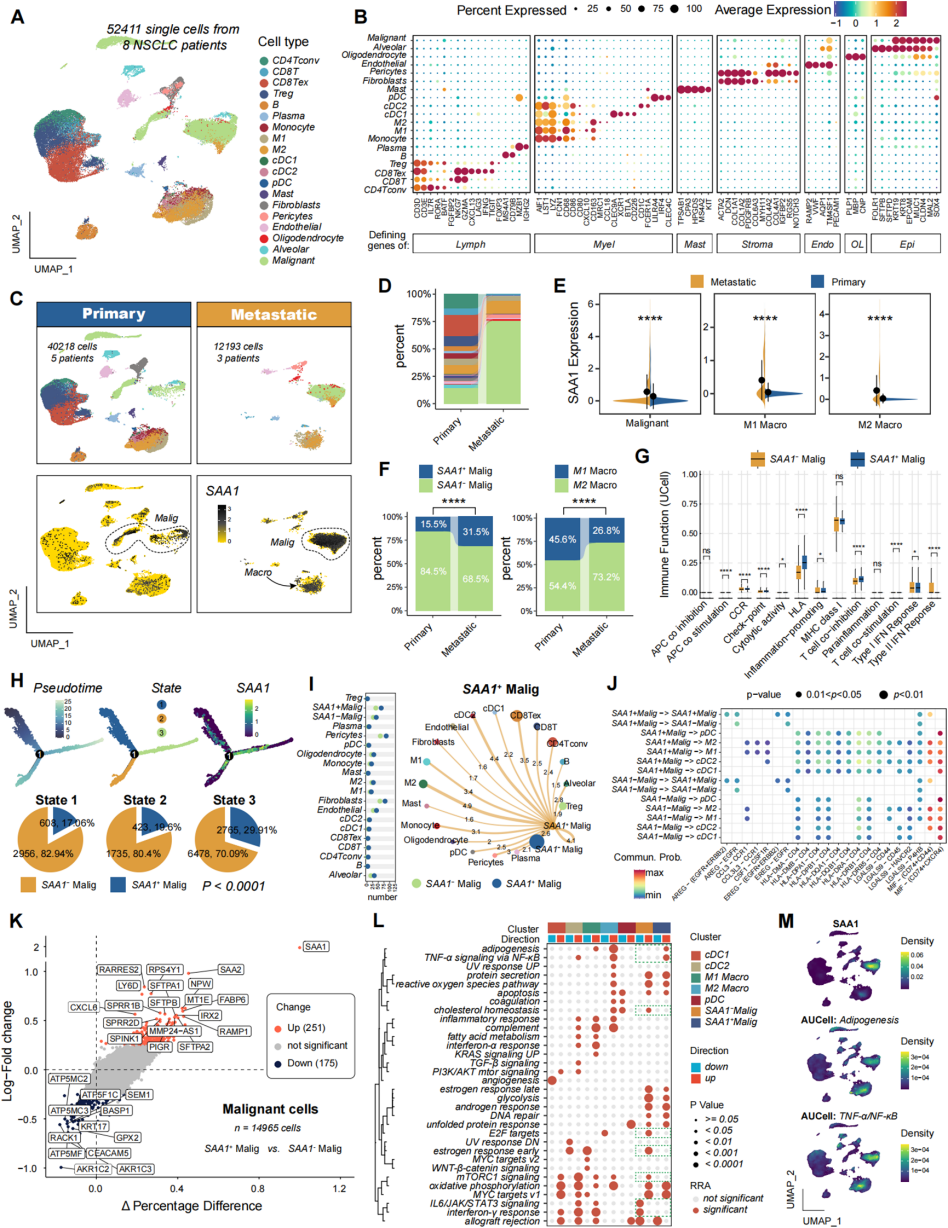
Single-cell transcriptomic profiling and analysis of SAA1+ malignant cells within NSCLC microenvironment. **(A)**, UMAP representation of 52,411 single cells from 8 NSCLC patients. **(B)**, Dot plot showing the percentage of cells expressing specific markers across different cell types, with color intensity indicating average expression levels. **(C)**, UMAP visualization of primary (the upper-left panel) and metastatic (the upper-right panel) cancer samples, highlighting the distribution of SAA1 expression in malignant and myeloid cells. **(D)**, Stacked bar chart illustrating the proportion of different cell types derived from primary versus metastatic samples. **(E)**, Box plots comparing SAA1 expression levels in malignant cells, M1 macrophages, and M2 macrophages across primary and metastatic samples. **(F)**, Stacked bar plots depicting the relative abundance of SAA1^+^ vs. SAA1^−^ malignant cells and of M1 versus M2 macrophages across primary and metastatic samples. **(G)**, Box plots of UCell scores for 13 immune-related functional states across SAA1^+^ and SAA1^−^ malignant cells, indicating significant differences in immune phenotypes. **(H)**, Pseudotime trajectory analysis of malignant epithelial cells, revealing distinct cellular states and distribution of SAA1^+^ malignant cells along the trajectory. **(I)**, Intercellular communication analysis showing the number of ligand-receptor interactions between SAA1^+^ and SAA1^−^ malignant cells (the left panel), and the communication network between SAA1^+^ malignant cells and other cell types in the NSCLC tumor microenvironment (the right panel). The thickness of the lines and numbers represent the interaction strength, highlighting key interactions. **(J)**, Dot plot of ligand-receptor interaction probabilities between SAA1^+^ and SAA1^−^ malignant cells and other cell types. The size and color of the dots indicate the probability and significance of interactions. **(K)**, Volcano plot displaying average log fold changes and percentage differences of differentially expressed genes between SAA1^+^ and SAA1^−^ malignant cells, highlighting significant upregulated and downregulated genes. Markers with an absolute average log fold change (|avg_logFC|) greater than 0.5, a difference in the percentage of gene-expressing cells (pct_in − pct_out) greater than 0.1, and an adjusted p-value (p_val_adj) less than 0.05 were labeled. **(L)**, Consensus hallmark pathway enrichment analysis of clusters. **(M)**, UMAP plots showing SAA1 expression density and AUCell scores for adipogenesis and TNF-α signaling via NF-κB pathways across the cell populations. Statistical significance. *P < 0.05, ****P < 0.0001; ns, not significant.

To investigate the relationship between SAA and the tumor immune microenvironment, we first performed a comprehensive bioinformatic analysis of lung adenocarcinoma (LUAD) cohorts (**Figures 1A, B, D, K**). SAA was found to be significantly upregulated in LUAD malignant populations compared to other cell types (**Figure 1C**) and interestingly the percentage of SAA^hi^ subpopulation was higher in malignant and macrophage from metastatic samples compared to primary tumors (**Figures 1C, E, F**). Among tumors, those with high SAA expression exhibited distinct immune-related transcriptional profiles. Immune function analysis revealed that SAA^hi^ populations correlates with the differential expression of numerous inflammatory genes including antigen presentation genes (**Figure 1G**). Next, pseudotime trajectory analysis of malignant epithelial cells, revealing distinct cellular states and distribution of SAA^hi^ malignant cells along the trajectory (**Figure 1H**). The Intercellular communication analysis and ligand-receptor interaction analysis both indicate strong interaction between SAA^hi^ malignant subpopulations and different types of APCs (**Figures 1I, J**). Gene set enrichment analysis further demonstrated that SAA^hi^ malignant populations were significantly enriched for pathways associated with inflammatory responses (such as TNF-α/NF-κB signaling) (**Figures 1K–M**). Collectively, these findings indicate that SAA signal in LUAD is linked to an immunosuppressive tumor immune landscape and correlates with antigen presenting populations in the tumor microenvironment.

### SAA restricts the differentiation of effector T cells

**Figure 2:**
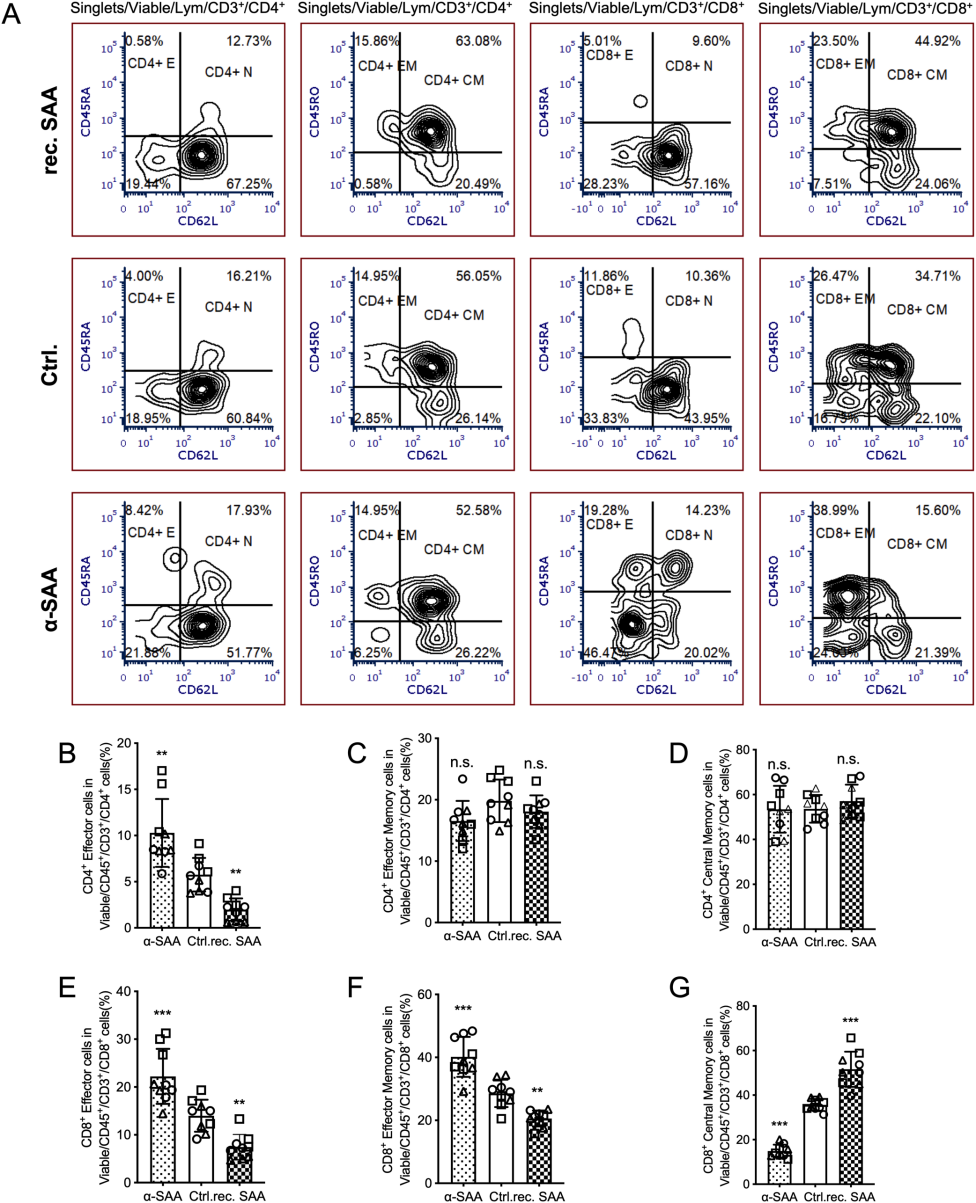
SAA exposure alters T cell subset distribution in a tumor organoid co-culture. **(A)**, Representative flow cytometry gating for T cells in 3 independent *ex vivo* autologous patient-derived organoids–T cell co-culture. **(B, E)**, The percentage of CD8^+^ and CD4^+^ effector T cells (CD45RA+/CD62L-) among viable CD45^+^CD3^+^ T cells in the co-culture under three conditions: control (Ctrl.), recombinant SAA (rec. SAA) treatment, or SAA-neutralizing antibody (α-SAA) treatment. **(C, F)**, The percentage of CD8^+^ and CD4^+^ effector memory T cells (CD45RO+/CD62L-) in the viable CD45^+^CD3^+^ T cell population for each treatment group. **(D, G)**, The percentage of CD8^+^ and CD4^+^ central memory T cells (CD45RO+/CD62L+) in the viable CD45^+^CD3^+^ T cell population under each condition. Statistical significance; **P < 0.01, ***P < 0.001; ns, not significant. Each assay was performed in triplicate.

To explore the effect of SAA on T cell functional differentiation, we established an *ex vivo* autologous T cell-tumor organoid co-culture system [as we reported in Wang et al., 2023, *Cell Death & Dis.* (16)] in which T lymphocytes were exposed to different SAA-related treatments, including recombinant SAA protein, control, and SAA-neutralizing antibody (α-SAA). Flow cytometry was used to assess the proportion and phenotype of T cell subsets (**Figure 2A**).

The results showed that treatment with recombinant SAA protein significantly reduced the proportion of CD4^+^ and CD8^+^ effector T cells, while markedly increasing the proportion of CD8^+^ central memory T cells (**Figures 2B–G**). These findings suggest that SAA protein may inhibit the generation of effector phenotypes in the tumor microenvironment, whereas neutralization of SAA enhances the effector response and suppresses memory cell formation. Additionally, there were no significant differences among groups in the proportions of CD4^+^ effector memory and central memory T cells (**Figures 2C, D**), while the proportion of CD8^+^ effector memory T cells was higher in the Ctrl. and rec. SAA groups compared to the α-SAA group (**Figure 2F**). Collectively, these results indicate that SAA protein plays an important role in regulating T cell subset differentiation within the tumor organoid co-culture system, potentially by modulating the balance between effector and memory T cell phenotypes, thereby influencing anti-tumor immune responses.

### SAA limits dendritic cell maturation

**Figure 3:**
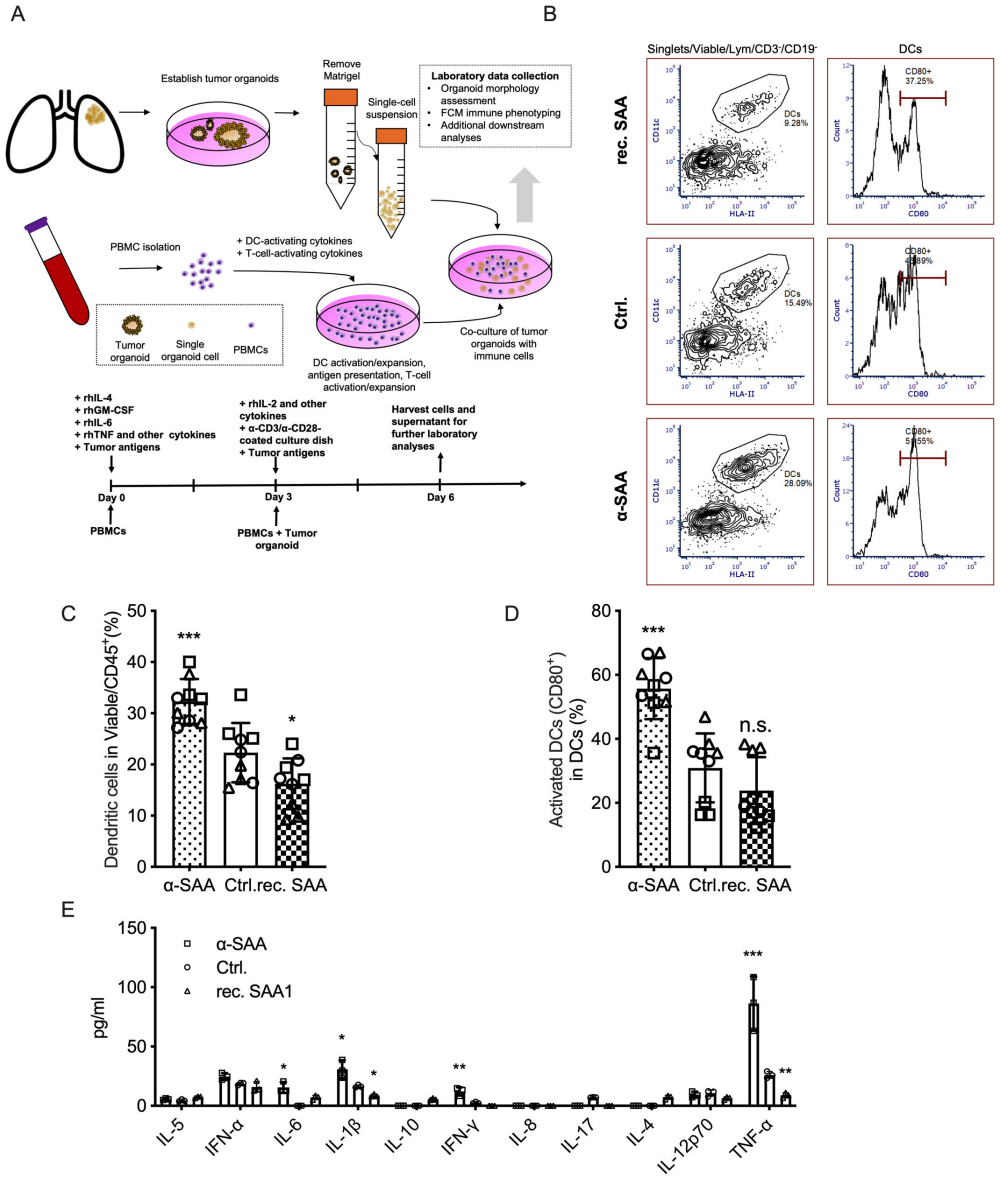
SAA suppresses dendritic cell expansion and activation. **(A)**, Schematic of 3 independent *ex vivo* co-culture experiment combining human adherent PBMCs and autologous lung tumor organoids. **(B)**, Flow cytometry gating for identifying DCs in the co-culture. **(C)**, The percentage of DCs (CD45^+^CD3^−^CD19^−^, phenotypically defined as DCs) among total viable CD45^+^ cells in the co-culture for each treatment. **(D)**, The percentage of activated DCs (CD80^+^ cells) within the gated DC population under each treatment. **(E)**, After cell culture, the supernatants were collected for cytokine measurement using cytometric beads array. Statistical significance: *P < 0.05, **P < 0.01, ***P < 0.001; ns, not significant. Each assay was performed in triplicate.

To further evaluate the impact of SAA on dendritic cell (DC) function in a physiologically relevant context, we established an *ex vivo* co-culture system using adherent human PBMCs and tumor organoids (**Figure 3A**). Flow cytometry gating strategies for identifying DCs and their activation status are shown in **Figure 3B**. Quantitative analysis revealed that recombinant SAA treatment significantly decreased the proportion of DCs among viable CD45^+^ cells compared to the control group, whereas α-SAA treatment led to an increase in DC frequency (**Figure 3C**). Moreover, the proportion of activated DCs (CD80^+^) within the DC population was markedly reduced by SAA exposure, while blockade of SAA with α-SAA restored DC activation to control or higher levels (**Figure 3D**). And for cell culture cytokine measurements, found that the SAA signaling significantly influenced the releasing of many T cell maturation/polarization and anti-tumor immunity related cytokines including, IL-1β, IFN-γ, TNF-α, IL-6 etc. (**Figure 3E**). These results demonstrate that SAA suppresses both the expansion and activation of DCs in the tumor organoid–PBMC co-culture system, highlighting a key immunosuppressive role of SAA in the tumor microenvironment.

### SAA suppresses macrophage activation and favors M2 polarization

**Figure 4:**
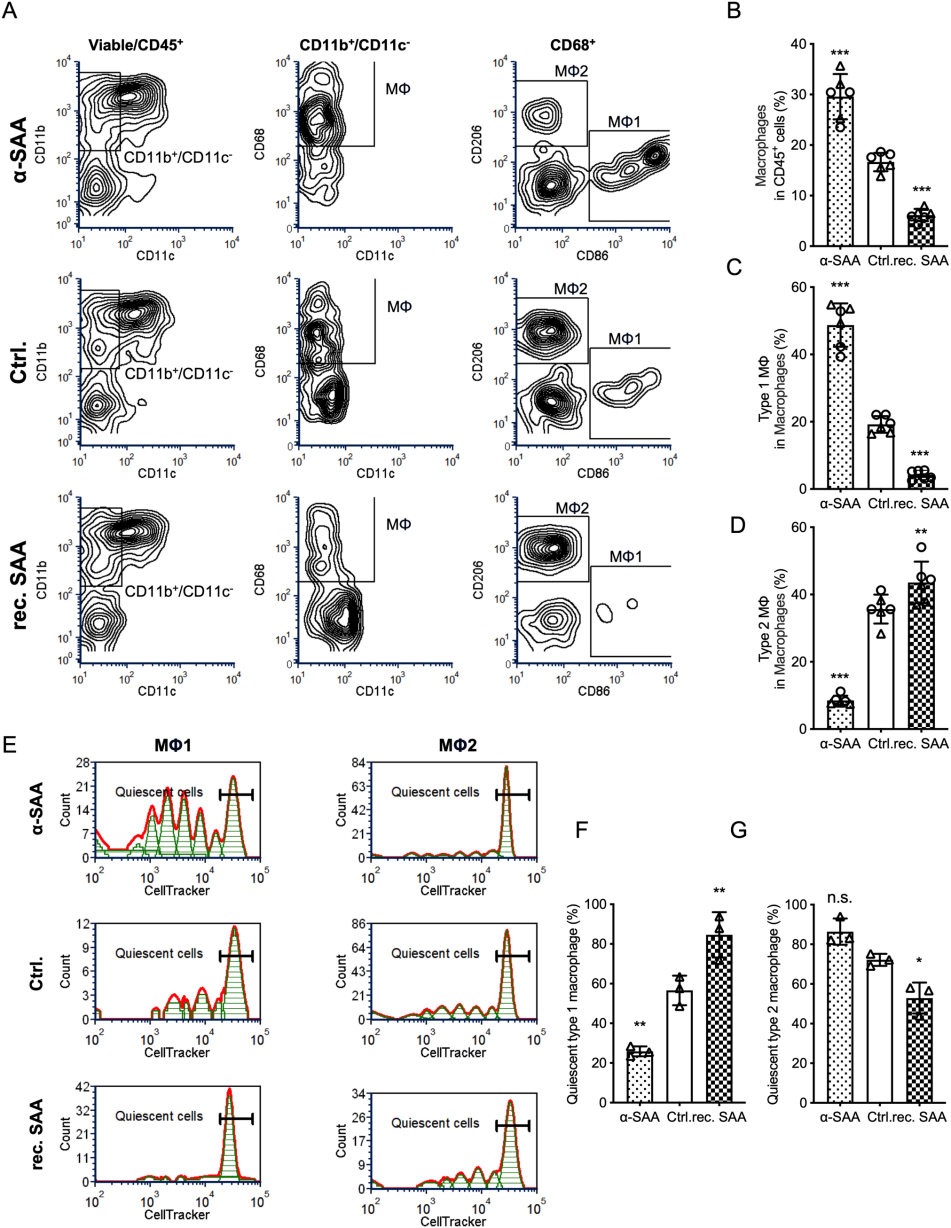
SAA suppresses macrophage activation and favors M2 polarization. **(A)**, Representative flow cytometry plots of human PBMC-derived macrophages (from healthy donors) after 5-day M-CSF treatment, showing the macrophage population among viable CD45^+^ cells under each condition (Ctrl., rec. SAA, α-SAA). **(B)**, The percentage of macrophages among viable CD45^+^ cells in culture for each treatment. **(C, D)**, The polarization state of the macrophages, **(C)** shows the percentage of M1 macrophages among total macrophages under each condition, and **(D)** shows the percentage of M2 macrophages among total macrophages. **(E)**, CellTracker proliferation assay results illustrating the cell division profile of macrophage subsets. Overlayed histograms of CellTracker dye dilution (division tracking) highlight the population of undivided (quiescent) macrophages. **(F, G)**, Bar graphs quantifying the fraction of quiescent (undivided, dye-retaining) cells in the M1 subset **(F)** and M2 subset **(G)** under each treatment condition. Statistical significance: *P < 0.05, **P < 0.01, ***P < 0.001; ns, not significant. Each assay was performed in triplicate.

To investigate the effect of SAA on macrophage polarization, human PBMCs were first differentiated into macrophages by culturing adherent monocytes with M-CSF for 5 days. The resulting macrophages were then treated with recombinant SAA, control, or SAA-neutralizing antibody (α-SAA), in the presence of tumor antigens and a low concentration of LPS to mimic a physiologically relevant activation environment. Flow cytometric analysis was performed to assess the proportion and polarization status of macrophages based on CD86 (M1 marker) and CD206 (M2 marker) expression (**Figure 4A**).

The overall proportion of macrophages among viable CD45^+^ cells decreased in the recombinant SAA group, while α-SAA treatment increased macrophage numbers compared to control (**Figure 4B**). Further analysis revealed that SAA exposure resulted in a significant reduction in the proportion of CD86^+^ (M1) macrophages (**Figure 4C**) and a corresponding increase in CD206^+^ (M2) macrophages (**Figure 4D**). Consistent with this shift, a CellTracker based proliferation assay also revealed that SAA-treated cultures were enriched with quiescent (cells undergone few divisions) M1 macrophages and less quiescent M2 macrophages while α-SAA restricted the distribution of quiescent M1 macrophages (**Figures 4E–G**). These findings demonstrate that SAA suppresses macrophage activation and skews macrophage polarization toward an immunosuppressive M2 state in the presence of tumor antigens.

### SAA restricts T cell infiltration *in vivo*

**Figure 5:**
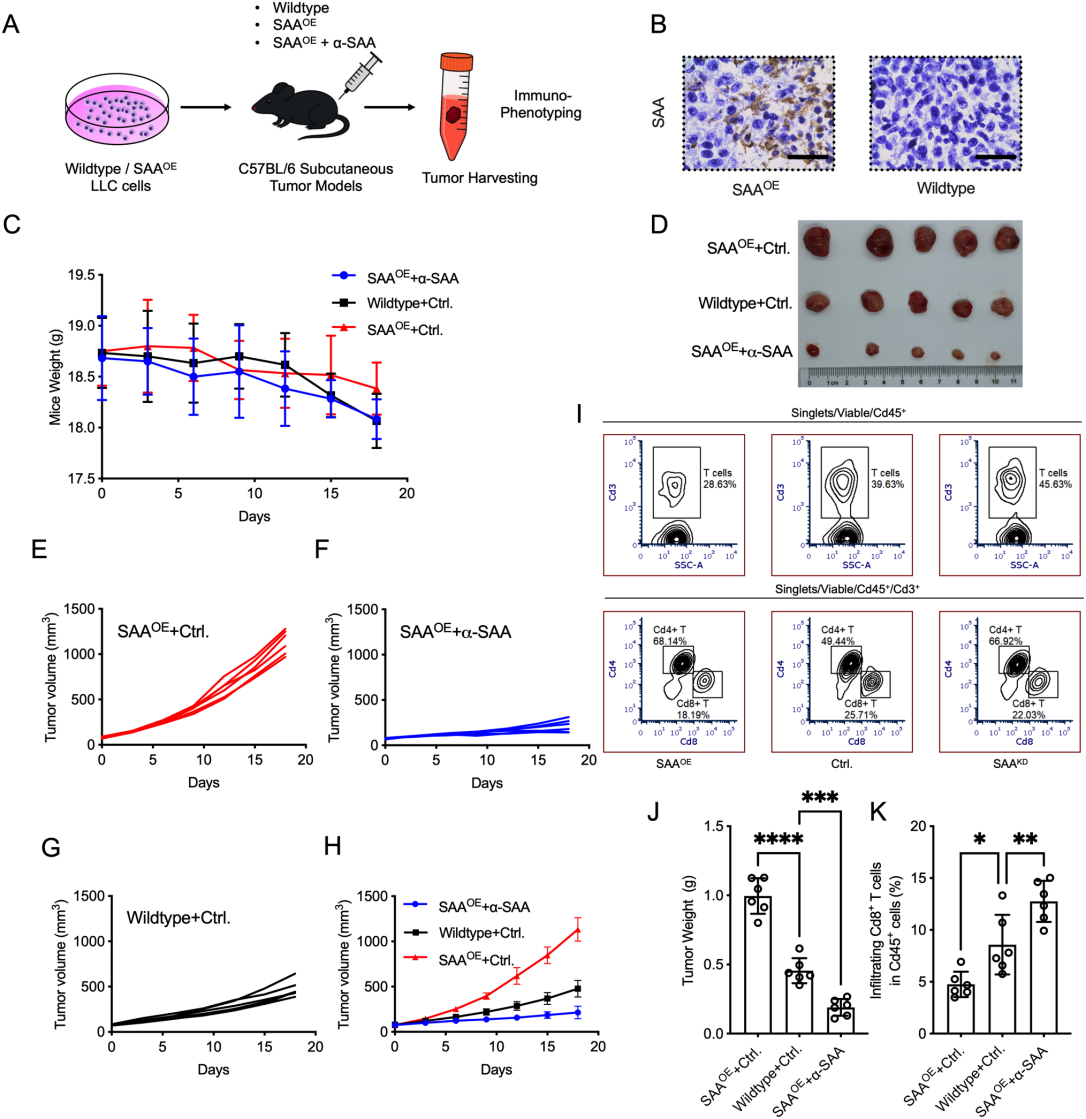
SAA expression enhances tumor growth *in vivo*. **(A)**, Schematic of the *in vivo* lung tumor model. C57BL/6 mice were injected subcutaneously with LLC lung carcinoma cells that were either SAA-overexpressing (SAA^OE^) or vector control (Wildtype). Mice in all groups received α-PD-1 antibody immunotherapy (three doses: on the day of tumor implantation, and on days 7 and 14). For treatment, mice received SAA neutralization antibody (α-SAA) or isotype control (Ctrl.) on the day of tumor implantation, and on days 7 and 14. **(B)**, Representative IHC staining of human SAA in SAA^OE^ and wild-type tumors (100µm scale bar). **(C)**, Line graph of mouse body weight over time for each group. **(D, J)**, Tumors and the final tumor weights at the experimental endpoint for each group. **(E–H)**, Tumor growth curves showing tumor volume (mm³) measured for each group. **(I, K)**, Representative flow cytometry plot illustrating the gating of tumor infiltration of T populations, the percentage of tumor infiltration Cd8+ T cells in Cd45+ cells. Statistical significance: *P < 0.05, **P < 0.01, ***P < 0.001, ****P < 0.0001. Each assay was performed in triplicate.

To assess the role of SAA in tumor progression and anti-tumor immunity *in vivo*, we established a subcutaneous tumor model in C57BL/6 mice using LLC cells with h.SAA1 overexpression [SAA^OE^, considering that human and mouse SAA proteins are highly homologous (32)], or vector control (Wildtype) (**Figures 5A, B**). Mice received 3 doses of PD-1 antibody and SAA neutralization antibody (α-SAA)/isotype control (Ctrl.) treatment on the day of tumor implantation, 7 days and 14 days. Throughout the experiment, Mice body weight was stable across all groups (**Figure 5C**). At the experimental endpoint, tumor weight was highest in the SAA^OE^ group and lowest in the SAA^OE^ + α-SAA group (**Figure 5D**). Tumor growth monitoring revealed that mice bearing SAA^OE^ tumors exhibited significantly increased tumor volume compared to the control group, while in SAA^OE^ + α-SAA treatment group, tumors grew substantially slower (**Figures 5E–H**). Flow cytometry analysis of tumor-infiltrating immune cells demonstrated that α-SAA significantly increased the proportion of T cells and Cd8^+^ T cells within the tumor microenvironment, whereas SAA overexpression reduced T cells and Cd8^+^ T cell infiltration (**Figures 5I–K**). These results indicate that SAA promotes tumor growth and suppresses anti-tumor immune responses by limiting the recruitment of cytotoxic T lymphocytes *in vivo*, and α-SAA treatment have translational potential.

### SAA modulates the differentiation and cytotoxic activity of tumor-infiltrating T cells *in vivo*

**Figure 6:**
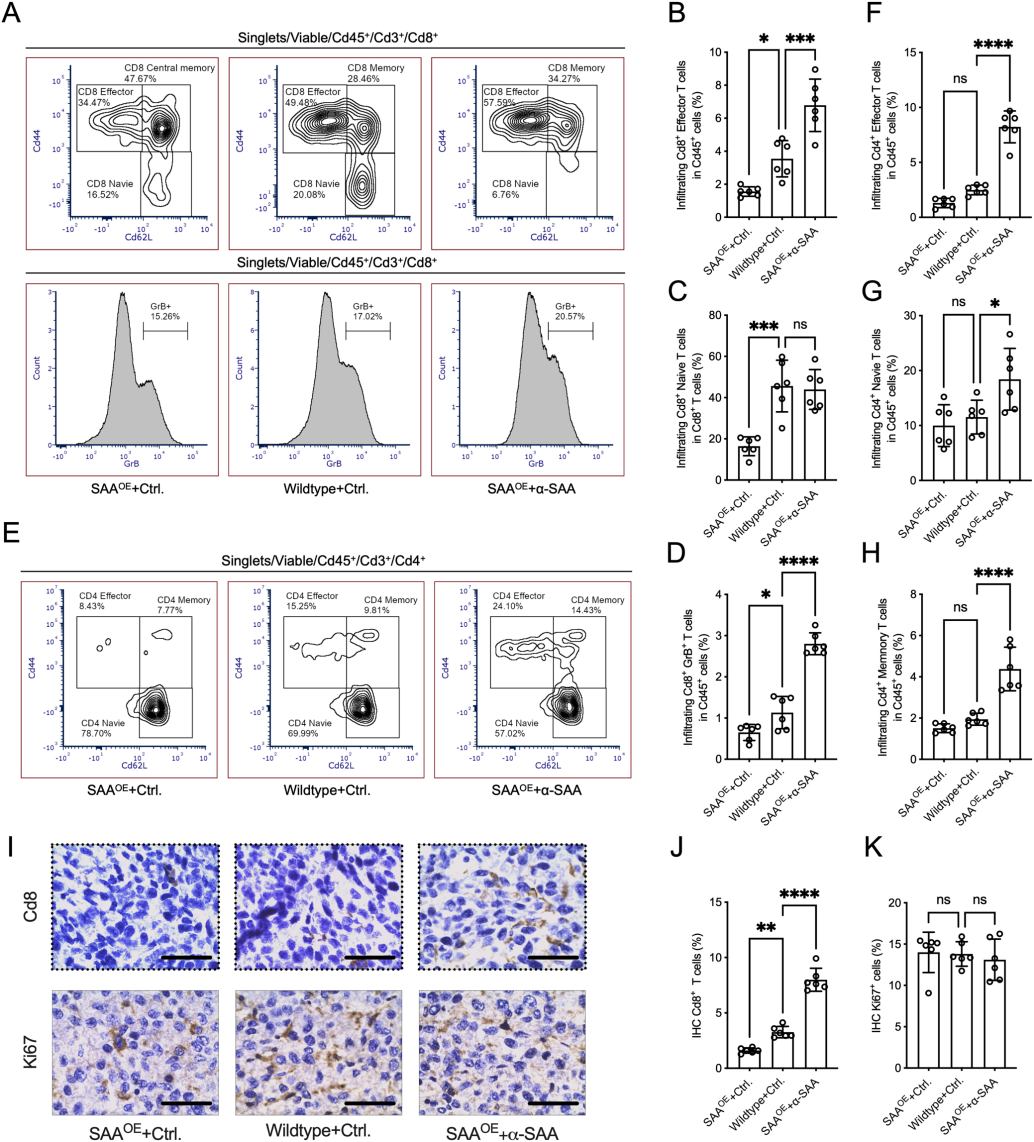
SAA limits cytotoxic T cell accumulation and activation in tumors. **(A)**, Representative flow cytometry plot illustrating the gating of effector, memory and naive T cell subsets among tumor-infiltrating Cd8^+^ T cells. **(B–D)**, The percentage of Cd8^+^ effector T cells (CD44^+^CD62L^−^), naive T cells (CD44^−^CD62L^+^) and Granzyme B^+^ cytotoxic T cells among total viable Cd45^+^ cells in the tumors of each group. **(E)**, Representative flow cytometry plot illustrating the gating of effector, memory and naive T cell subsets among tumor-infiltrating Cd4^+^ T cells. **(F–H)**, The percentage of Cd4^+^ effector T cells (Cd44^+^Cd62L^−^), naive T cells (Cd44^−^Cd62L^+^) and Cd4^+^ memory T cells (Cd44^+^CD62L^+^) among total viable tumor Cd45^+^ cells. **(I)**, Representative IHC staining of Cd8 and Ki67 in wildtype, SAA^OE^, and SAA^OE^ + α-SAA tumors. **(J, K)**, The percentage of Cd8+ and Ki67+ cells among nucleated cells in IHC analysis. Statistical significance: *P < 0.05, **P < 0.01, ***P < 0.001, ****P < 0.0001; ns, not significant. Each assay was performed in triplicate.

To further characterize the effect of SAA on tumor-infiltrating T cell subsets, we performed flow cytometric analysis of single-cell suspensions prepared from tumors harvested in animal experiments. As shown in **Figures 6A–C**, the proportion of Cd8^+^ effector T cells (Cd44^+^Cd62L^−^) among Cd45^+^ cells were significantly reduced in the SAA^OE^ group and increased in the SAA^OE^ + α-SAA treatment group, compared to Wildtype + Ctrl. treatment group. Conversely, the frequency of naïve Cd8^+^ T cells (Cd44^−^Cd62L^+^) within the Cd8^+^ T cell population was significantly higher in SAA^OE^ tumors but reduced in the SAA^OE^ + α-SAA treatment group. Analysis of cytotoxic activity revealed that the proportion of granzyme B^+^ Cd8^+^ T cells was lowest in the SAA^OE^ + Ctrl. treatment group and highest in the SAA^OE^ + α-SAA treatment group (**Figure 6D**). For Cd4^+^ T cells, SAA^OE^ + α-SAA treatment significantly expanded the effector, naïve and memory population (**Figures 6E–H**). In addition, IHC analysis revealed that SAA^OE^ tumors displayed markedly reduced Cd8^+^ T cell infiltration compared to wild-type tumors, whereas treatment with α-SAA restored Cd8^+^ cell infiltration (**Figures 6I, J**). And Ki67 staining showed no significant differences among groups, indicating that SAA doesn’t affects tumor cell proliferation (**Figures 6I, K**). These results indicate that SAA suppresses the accumulation and functional differentiation of Cd8^+^ and Cd4^+^ effector T cells within the tumor microenvironment, further supporting the immunosuppressive role of SAA as well as the clinical potential of α-SAA *in vivo*.

### SAA suppresses macrophage M1 polarization and maturation *in vivo*

**Figure 7:**
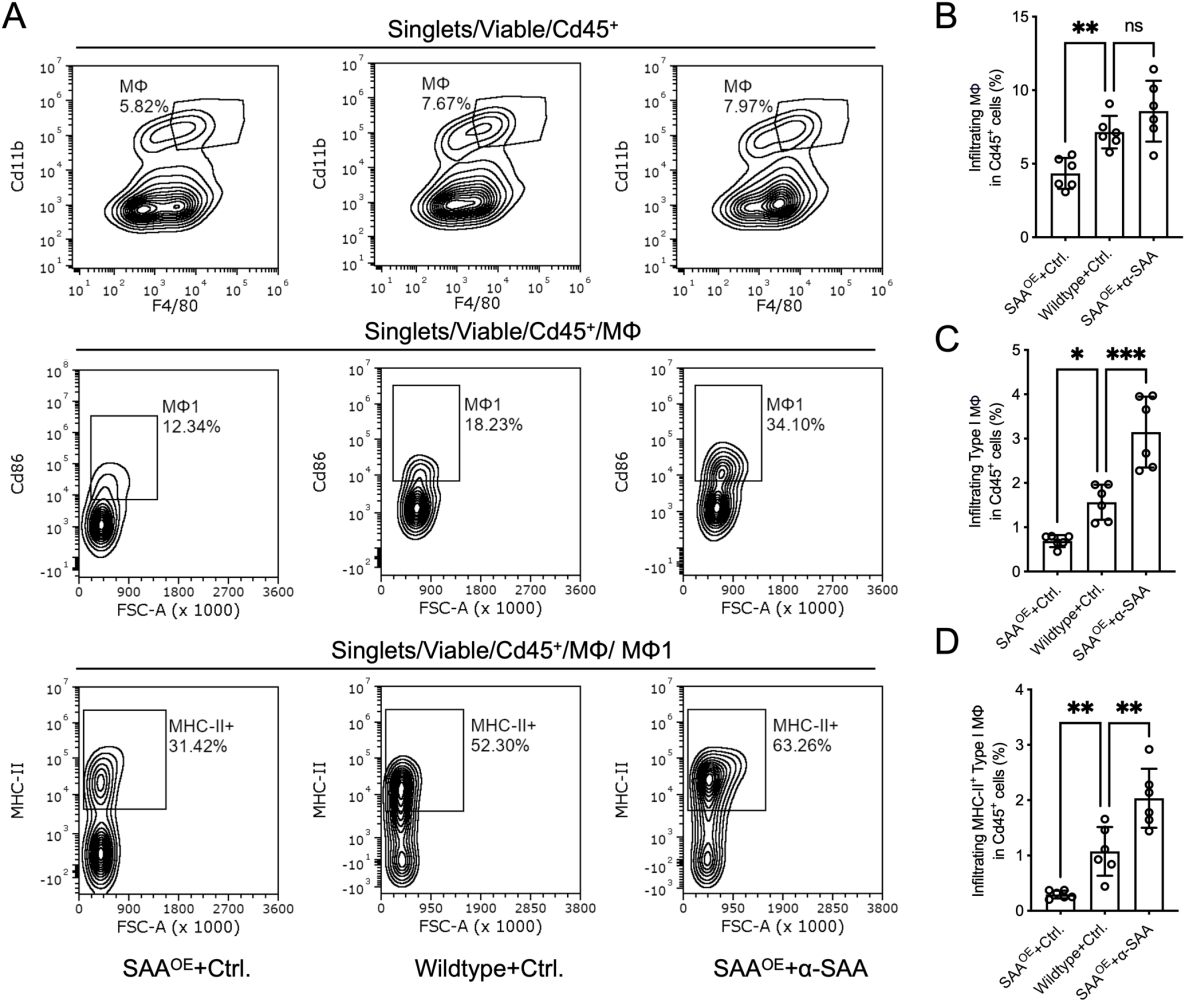
SAA suppresses macrophage infiltration and M1 antigen-presenting activation *in vivo*. **(A)**, Representative flow cytometry plot illustrating the gating of tumor-infiltrating total macrophages, M1 macrophages and activated M1 macrophages (MHC-II^+^). **(B–D)**, The percentage of tumor infiltrating total macrophages (Cd11b^+^/F4/80^+^), M1 macrophages (Cd86^+^ macrophages) and activated M1 macrophages (MHC-II^+^ M1 macrophages) in each group. Statistical significance: *P < 0.05, **P < 0.01, ***P < 0.001; ns, not significant. Each assay was performed in triplicate.

Given that macrophages are critical APCs responsible for initiating and sustaining T cell responses, and our prior results indicated that SAA impairs Cd8^+^ T cell activation *in vivo*, we hypothesized that SAA may exert its immunosuppressive effects by altering macrophage infiltration, activation, and antigen-presenting capacity within the tumor microenvironment. To address this, we performed detailed flow cytometric analysis of tumor-infiltrating macrophage populations in the animal model. As shown in **Figures 7A, B**, the overall proportion of tumor-infiltrating macrophages (Cd45^+^Cd11b^+^F4/80^+^) was significantly decreased in the SAA^OE^ + Ctrl. treatment group and increased in the SAA^OE^ + α-SAA treatment group, compared to SAA^WT^ + control treatment group. Analysis of macrophage polarization revealed that SAA^OE^ markedly reduced the frequency of M1 macrophages (**Figure 7C**), and this was accompanied by a further reduction in the subset of activated, MHC-II^+^ M1 macrophages (**Figure 7D**). In contrast, SAA^OE^ + α-SAA treatment promoted both M1 polarization and MHC-II upregulation. Additionally, the proportion of M2 macrophages was relatively increased in the SAA^OE^ group and reduced in the SAA^OE^ + α-SAA treatment group (**Figure 7D**). These findings demonstrate that SAA limits macrophage M1 polarization and activation, contributing to impaired T cell activation in tumors.

### SAA-conditioned macrophages impair T cell activation and cytotoxic function

**Figure 8:**
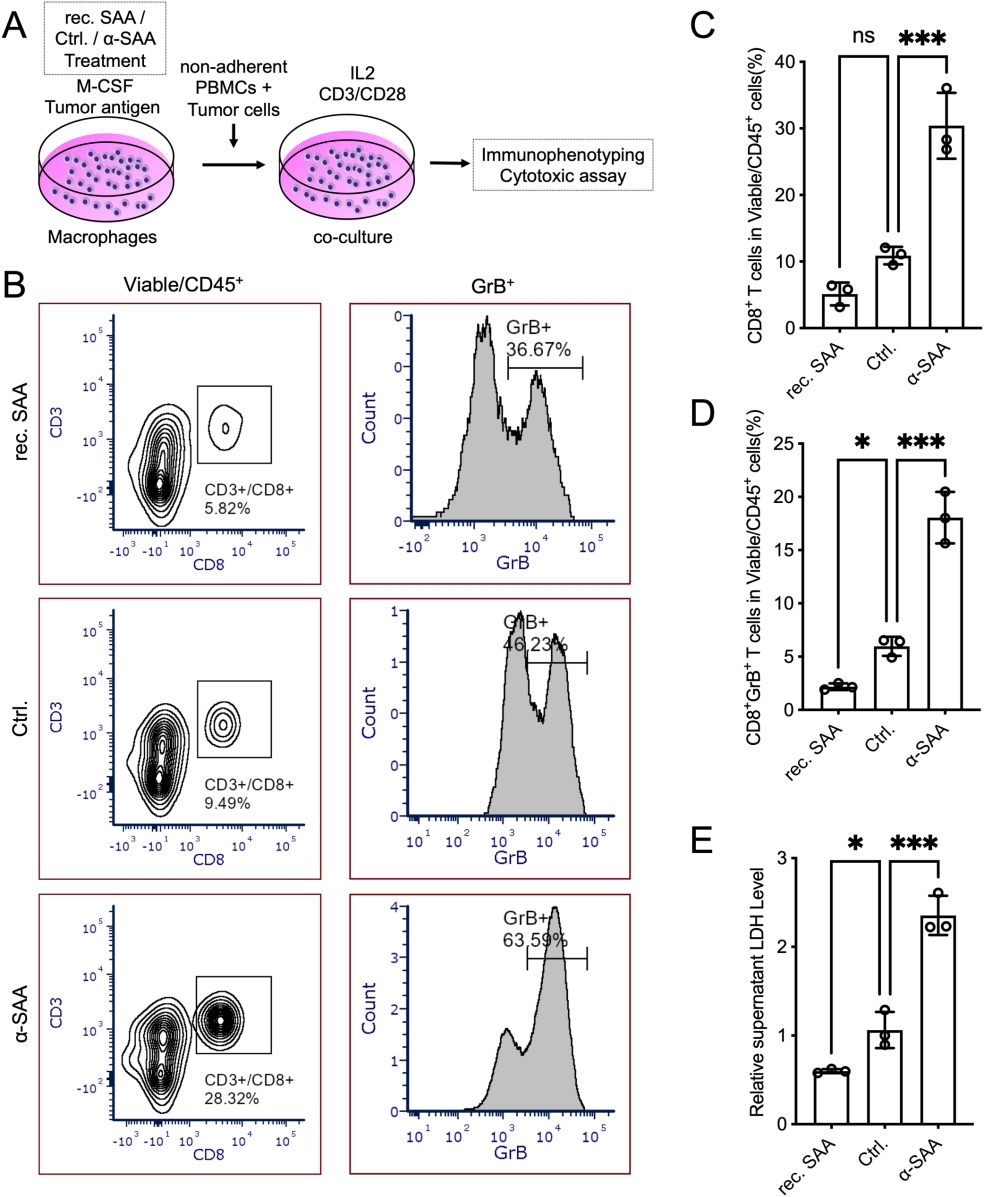
SAA-conditioned macrophages impair T cell activation and cytotoxicity in co-culture. **(A)**, Schematic of the macrophage–T cell co-culture assay. Human PBMC-derived macrophages (from healthy donors) were first pre-treated for 5 days with either control medium (Ctrl.), recombinant SAA (rec. SAA), or SAA-neutralizing antibody (α-SAA). In the second phase, these “conditioned” macrophages were co-cultured with autologous non-adherent PBMCs and tumor cells in the presence of IL-2 and anti-CD3/anti-CD28 stimulation to activate T cells. After 3 days of co-culture, T cell activation and tumor cell killing were evaluated by flow cytometry and supernatant LDH cytotoxicity assays, respectively. **(B)**, Representative flow cytometry plots from the co-cultures, illustrating the gating and frequency of CD8^+^ T cells among viable cells. **(C, D)**, The percentage of CD8^+^ T cells and Granzyme B^+^ cytotoxic T cells in the co-culture for each macrophage pre-treatment condition in the co-culture. **(E)**, The LDH release from tumor cells in the co-culture supernatants for each condition. Statistical significance: *P < 0.05, ***P < 0.001; ns, not significant. Each assay was performed in triplicate.

The previous results provided correlational evidence that SAA influences macrophages, T cells, and anti-tumor immunity independently, prompting us to hypothesize that SAA modulates anti-tumor immunity by altering macrophage function and subsequent T-cell activation. To directly validate SAA-macrophage-T cells axis influence anti-tumor immune responses, we designed the following functional experiments. Human PBMC-derived macrophages were first pre-treated with recombinant SAA (rec. SAA), control, or SAA-neutralizing antibody (α-SAA) for 5 days during the pre-treatment phase. These conditioned macrophages were then co-cultured with autologous non-adherent PBMCs and tumor cells in the presence of IL-2 and α-CD3/CD28 stimulation to activate T cells (**Figure 8A**). After 3 days, co-cultures containing SAA-conditioned macrophages showed a significantly lower proportion of CD8^+^ T cells compared to controls (**Figures 8B, C**). Likewise, the frequency of Granzyme B^+^ cytotoxic T cells was markedly reduced in the SAA-conditioned group, indicating impaired T cell cytotoxic activation (**Figure 8D**). Consistent with these findings, SAA-treated macrophages led to diminished tumor cell killing, as evidenced by significantly lower LDH release in the co-culture supernatant relative to controls (**Figure 8E**). Importantly, macrophages pre-treated with the SAA-neutralizing antibody restored T cell activation and cytotoxic function: in co-cultures with α-SAA-treated macrophages, CD8^+^ T cell percentages, Granzyme B^+^ T cell frequencies, and LDH release levels were all higher than in the SAA-treated condition (approaching or exceeding control levels) (**Figures 8C–E**). This reversal by α-SAA highlights the specific role of SAA in mediating the suppressive effects. In summary, we demonstrate that SAA-conditioned macrophages significantly impair CD8^+^ T cell activation and their anti-tumor activity, while neutralizing SAA can alleviate this immunosuppressive impact.

### SAA-CD36 interaction restricts macrophage maturation

**Figure 9:**
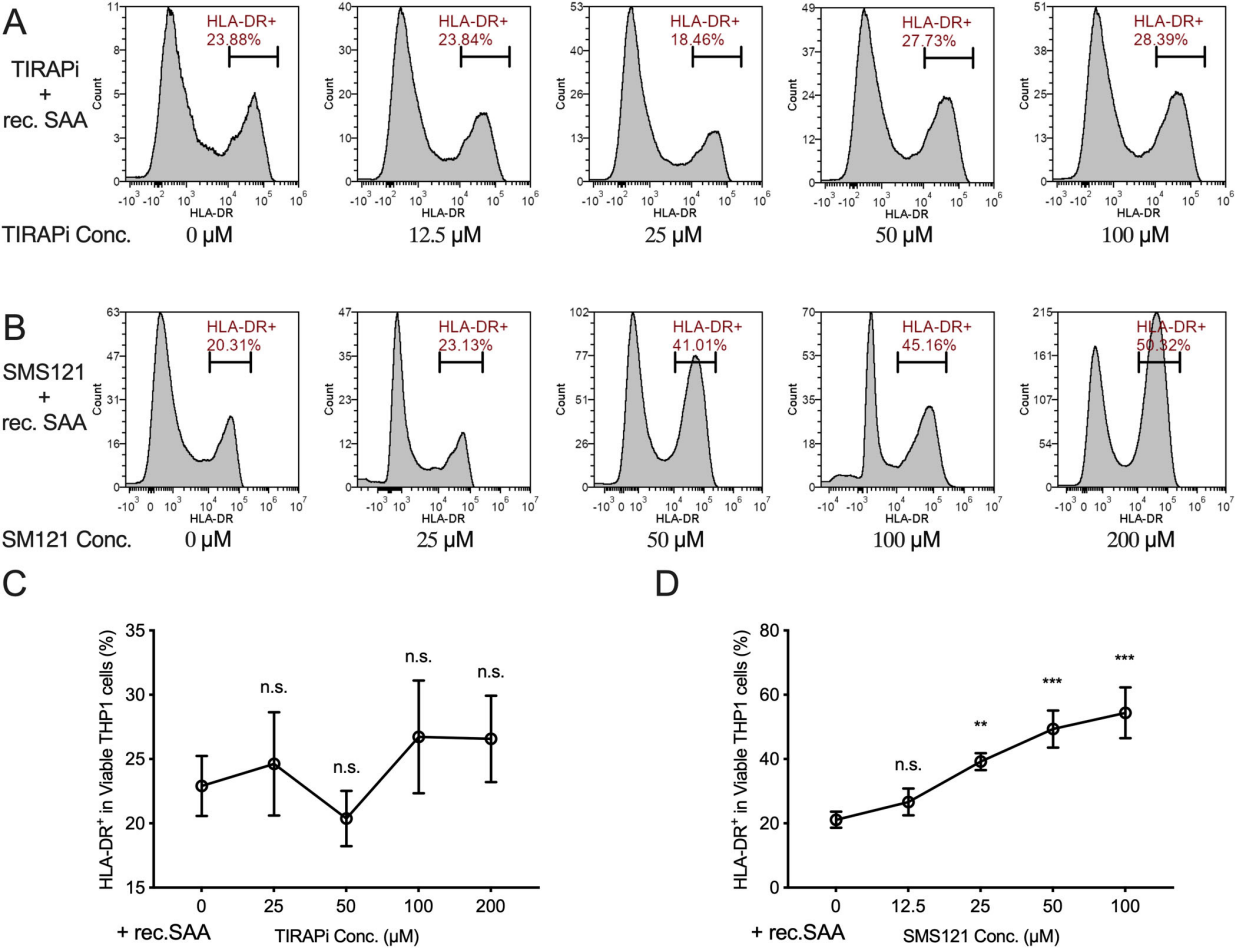
SAA restricts macrophage maturation by interacting with CD36. **(A, B)**, Representative flow cytometry plots from the inhibitor testing in THP1 cells cultured with rec. SAA. **(C, D)**, Percentage of HLA-DR^+^ THP1 cells with increasing TIRAP inhibitor (0, 25, 50, 100, 200 µM) and SMS121/CD36 inhibitor (0, 12.5, 25, 50, 100 µM) concentrations. Statistical significance: **P < 0.01, ***P < 0.001; ns, not significant. Each assay was performed in triplicate.

To further clarify the receptor pathway through which SAA inhibits macrophage maturation, we introduced a concentration gradient of the TLR2/4 pathway inhibitor TIRAPi or the CD36 inhibitor SMS121 under constant recombinant SAA stimulation and measured the proportion of HLA-DR^+^ THP1 derived macrophages (with M-CSF). The results showed that the TIRAP inhibitor did not markedly reverse the inhibitory effect of SAA on HLA-DR expression (**Figures 9A, C**), whereas with increasing concentrations of SMS121, the proportion of HLA-DR^+^ cells gradually recovered, and the SAA-mediated inhibitory effect was alleviated in a dose-dependent manner (**Figures 9B, D**). These findings indicate that blocking the CD36 pathway can antagonize the inhibitory effect of SAA on macrophage maturation, suggesting that SAA primarily signals through the scavenger receptor CD36 rather than TLR2/4 to inhibit macrophage maturation and activation. The above results further support the key role of the SAA–CD36 axis in the regulation of macrophage maturation and activation.

## Discussion

In current work, we found that tumors with elevated SAA expression exhibited distinct immune-related transcriptional profiles, including the upregulation of inflammatory cytokines and the downregulation of genes associated with antigen presentation and T-cell activation. The following experiments provide a deeper mechanistic understanding of how SAA orchestrates immune evasion within the tumor microenvironment, specifically through modulation of APC function and T cell activation.

We observed that SAA significantly impaired the activation and maturation of DCs and macrophages. These APCs are critical for initiating and maintaining cytotoxic T lymphocyte (CTL) responses through effective antigen presentation and co-stimulation (5, 6). Our *ex vivo* co-culture models demonstrated that exposure to recombinant SAA reduced DCs maturation markers such as CD80 and MHC-II, resulting in compromised antigen-presenting capability. Similarly, macrophages exposed to SAA exhibited reduced M1 polarization and proliferation, characterized by lower CD86, MHC-II expression and higher percentages of quiescent macrophages, and skewed toward an immunosuppressive M2 phenotype (CD206^+^). These findings align well with previous reports that tumors manipulate macrophage polarization and DC maturation to evade immune surveillance (6, 15, 32, 33).

Consistent with *ex vivo* findings, our *in vivo* data revealed that tumors overexpressing SAA (SAA^OE^) grew faster and displayed reduced infiltration and activation of CD8^+^ T cells, whereas SAA^OE^ tumors with α-SAA treatment showed the opposite phenotype. Specifically, we found a marked suppression of effector Cd8^+^ T cell differentiation (Cd44^+^Cd62L^−^) and decreased cytotoxic function (granzyme B^+^ cells) in the presence of high SAA. These results suggest that SAA-mediated APC dysfunction directly curtails the activation and cytotoxic potential of tumor-infiltrating Cd8^+^ T cells. Similar immunosuppressive strategies are observed with other tumor-secreted factors such as IL-10, VEGF, G-CSF and IL6, which likewise limit APC activation and T cell-mediated anti-tumor immunity (9, 15, 31, 34, 35). Last, in line with our findings, functional co-culture assays provided direct evidence that SAA-conditioned macrophages markedly suppress CD8^+^ T cell priming and cytotoxic activity. Macrophages exposed to SAA curtailed the expansion of CD8^+^ T cells and the generation of Granzyme B–expressing effector T cells, resulting in significantly impaired tumor cell killing.

Interestingly, Stone et al. recently reported that systemic SAA, induced via IL-6–STAT3 signaling, can impair DC function in pancreatic cancer, leading to decreased T cell infiltration and poor patient prognosis (15). Our findings in lung cancer confirm and extend this mechanism by demonstrating SAA-driven macrophage dysfunction alongside DCs impairment, highlighting the broader immunosuppressive role of SAA across cancer types. Moreover, the observation that neutralizing SAA with an antibody restored APC maturation and T cell activation points toward therapeutic opportunities. Indeed, targeting tumor-associated macrophages and DCs to enhance antigen presentation is currently gaining attention as a complementary strategy to checkpoint blockade (34). Mechanistically, here we report that the immunosuppressive phenotype induced by SAA on APCs is dependent on CD36 receptor, CD36 mediates lipid uptake (e.g., oxLDL, fatty acids) in macrophages, modulates immunometabolism and inflammation, and promotes immunosuppressive phenotypes in antigen-presenting cells within chronic inflammation and tumor microenvironments (36), this finding is consistent with literature reports: SAA can act on multiple receptors, with CD36 serving as an important signaling pathway; activation of the CD36 receptor often drives macrophages toward an immunosuppressive M2 phenotype, accompanied by decreased antigen-presenting capacity and inhibition of T cell activation (37, 38). Therefore, combining SAA-neutralizing strategies with existing immunotherapies, such as anti-PD-1 therapy, might improve clinical outcomes in patients with SAA rich tumors.

Clinically, elevated circulating SAA levels have been correlated with poor prognosis in various cancers, including lung and pancreatic cancer, reinforcing its potential role as a prognostic biomarker for immunotherapy resistance (15, 32, 39–41). Our findings further underscore the therapeutic potential of SAA blockade, particularly in patients who do not respond adequately to current checkpoint inhibitors. The feasibility of targeting SAA signaling pathways (e.g., P2X7 receptor blockade) provides an additional therapeutic avenue worthy of exploration (16). While we have to note that, SAA−targeting strategies might be promising for facilitating anti−tumor immunity, but the nature of SAA is an acute−phase protein, which may pose translational challenges that warrant careful evaluation.

In conclusion, our study provides compelling evidence that tumor-derived SAA suppresses anti-tumor immunity by impairing APC activation and antigen presentation via CD36, thus hindering CD8^+^ T cell-mediated cytotoxic responses. These findings position SAA as a key immune regulator and potential therapeutic or biomarker target, highlighting new possibilities for enhancing the efficacy of cancer immunotherapy.

## Data Availability

The raw data supporting the conclusions of this article will be made available by the authors, without undue reservation.
