# Supplementary material for: SAA restricts T cell mediated anti-tumor immunity by limiting antigen presentation in lung cancer
Source: Front Immunol. 2025 Dec 19;16:1735479. doi: 10.3389/fimmu.2025.1735479 (PMC12757294; doi:10.3389/fimmu.2025.1735479)
Supplement: Supplementary file 1 [file Table1.docx]

# **SAA restricts T cell mediated anti-tumor immunity by limiting antigen presentation**

*Mei Huang ^1,2^, *Run Shi ^3^, Cong Xu ^1^, Yihan Zhang ^1^, Xiaoyue Du ^1^, Shaodi Wen ^1^, Chunbin Wang ^2^, Feng Jiang ^4^, Guoren Zhou ^1^ ^#^Xin Wang ^1^, ^#^Bo Shen ^1,5,6^

1, Department of Oncology, Jiangsu Cancer Hospital & Jiangsu Institute of Cancer Research & The Affiliated Cancer Hospital of Nanjing Medical University

2, Department of Oncology, Yancheng Third People's Hospital

3, Department of Oncology, First Affiliated Hospital of Nanjing Medical University, Nanjing, China

4, Department of thoracic surgery, Jiangsu Cancer Hospital & Jiangsu Institute of Cancer Research & The Affiliated Cancer Hospital of Nanjing Medical University

5, Department of Oncology, Affiliated Huishan Hospital of Xinglin College, Nantong University, Wuxi Huishan District People's Hospital, Wuxi, China

6, Department of Oncology, Huai’an Hongze District People’s Hospital, Huaian, China

*These authors contribute equally to this work.

^#^Correspondent to:

Shen Bo: [shenbo987@njmu.edu.cn](mailto:shenbo987@njmu.edu.cn), Department of Oncology, Jiangsu Cancer Hospital & Jiangsu Institute of Cancer Research & The Affiliated Cancer Hospital of Nanjing Medical University, Baiziting 42, Nanjing, Jiangsu, China

Wang Xin: [xinwang@njmu.edu.cn](mailto:xinwang@njmu.edu.cn), Department of Oncology, Jiangsu Cancer Hospital & Jiangsu Institute of Cancer Research & The Affiliated Cancer Hospital of Nanjing Medical University, Baiziting 42, Nanjing, Jiangsu, China

### Table S1

| **Flow Cytometry reagent** |  | | |  |  |
| --- | --- | --- | --- | --- | --- |
| **Antibody** | **Species** | **Clone** | **Vender** | | |
| CD45 | Human | HI30 | Biolegend | | |
| CD3 | Human | OKT3 | Thermo Fisher | | |
| CD4 | Human | SK3 | Thermo Fisher | | |
| CD8 | Human | RPA-T8 | Biolegend | | |
| CD45RA | Human | HI100 | Thermo Fisher | | |
| CD45RO | Human | UCHL1 | Thermo Fisher | | |
| CD62L | Human | DREG-56 | Biolegend | | |
| CD19 | Human | HIB19 | Thermo Fisher | | |
| CD11c | Human | 3.9 | Thermo Fisher | | |
| HLA-DR | Human | LN3 | Biolegend | | |
| CD80 | Human | 2D10.4 | Thermo Fisher | | |
| CD11b | Human | C67F154 | Thermo Fisher | | |
| CD68 | Human | KP1 | Thermo Fisher | | |
| CD206 | Human | 19.2 | Thermo Fisher | | |
| CD86 | Human | BU63 | Thermo Fisher | | |
| GrB | Human / Mouse | GB11 | Biolegend | | |
| Cd45 | Mouse | 30-F11 | Biolegend | | |
| Cd3 | Mouse | 145-2C11 | Thermo Fisher | | |
| Cd4 | Mouse | GK1.5 | Biolegend | | |
| Cd8 | Mouse | 53-6.7 | Thermo Fisher | | |
| Cd44 | Mouse | IM7 | Thermo Fisher | | |
| Cd62L | Mouse | MEL-14 | Thermo Fisher | | |
| Cd11b | Mouse | M1/70 | Thermo Fisher | | |
| F4/80 | Mouse | BM8 | Thermo Fisher | | |
| Cd86 | Mouse | GL1 | Thermo Fisher | | |
| MHC-II | Mouse | M5/114.15.2 | Thermo Fisher | | |

### Table S2

| **Other reagent** | **Cat. No.** | **Vender** |
| --- | --- | --- |
| UltraComp eBeads™ Plus | 01-3333-42 | Thermo Fisher |
| RBC Lysis Buffer | 00-4333-57 | Thermo Fisher |
| Fixation / Permeabilization Concentrate | 00-5123-43 | Thermo Fisher |
| CD16 / CD32 Monoclonal Antibody (93) | 4-0161-82 | Thermo Fisher |
| Fixation / Permeabilization Concentrate | 00-5123 | Thermo Fisher |
| LIVE/DEAD cell viability Dye (488nm) | L34970 | Thermo Fisher |
| Fixable Viability Dye eFluor™ 506 | 65-0866-14 | Thermo Fisher |
| CellTracker Green CMFDA | C7025 | Thermo Fisher |
